# Headache, Fever, and Myalgias in an HIV-Positive Male with a History of Tuberculosis: Epstein–Barr Virus Aseptic Meningitis

**DOI:** 10.3390/tropicalmed8040191

**Published:** 2023-03-26

**Authors:** Loukas Kakoullis, Claudia Hentschel, Robert Colgrove

**Affiliations:** 1 Department of Internal Medicine, Mount Auburn Hospital, Cambridge, MA 02138, USA; 2 Harvard Medical School, Boston, MA 02138, USA; 3 Division of Infectious Diseases, Mount Auburn Hospital, Cambridge, MA 02138, USA

**Keywords:** HIV, aseptic meningitis, tuberculosis imaging, weeping willow sign, amphoric breath sounds, Epstein–Barr virus reactivation, aseptic meningitis

## Abstract

Background: We describe a case of EBV aseptic meningitis in a patient with HIV with an extensive history of prior infections and exposures. Detailed Case Description: A 35-year-old man with a history of HIV, syphilis, and partially treated tuberculosis presented with headache, fever, and myalgias. He reported recent exposure to dust from a construction site and had sexual contact with a partner with active genital lesions. An initial workup revealed mildly elevated inflammatory markers, significant pulmonary scarring from tuberculosis with a classic “weeping willow sign”, and lumbar puncture findings consistent with aseptic meningitis. An extensive evaluation was conducted to identify causes of bacterial and viral meningitis, including syphilis. Immune reconstitution inflammatory syndrome and isoniazid-induced aseptic meningitis were also considered based on his medications. EBV was ultimately isolated through PCR from the patient’s peripheral blood. The patient’s condition improved, and he was discharged on his home antiretroviral and anti-tuberculous treatment. Conclusion: Central nervous system infections represent unique challenges in patients with HIV. EBV reactivation can present with atypical symptoms and should be considered as a cause of aseptic meningitis in this population.

## 1. Introduction

Central nervous system (CNS) infections in immunocompromised patients represent a unique challenge, as in addition to the typical pathogens that cause them, this population is susceptible to a vast array of pathogens. Patients with HIV are a unique subset within this population, who are at increased risk of CNS infections by multiple pathogens, including *Cryptococcus neoformans*, *Mycobacterium tuberculosis*, and *Toxoplasma gondii*, as well as multiple other causes [1].

Epstein–Barr virus (EBV) is a known cause of aseptic meningitis, with a known predilection for immunocompromised hosts [2]. While CNS infection by EBV in HIV is well established, the typical presentation is that of primary CNS lymphoma [3]. We present a case of a patient with HIV presenting with symptoms consistent with a CNS infection and multiple exposures, who after an extensive workup was found to have aseptic meningitis due to EBV reactivation.

## 2. Detailed Case Description

A 35-year-old man presented to the emergency department with headache, fever, and myalgias. He reported one week of radiating frontal headaches and 3–4 days of subjective fever and myalgias, night sweats, and waking up with lower extremity muscle cramping at night. He also reported a 2.5 kg weight loss over the previous 2 weeks. He specifically denied any photosensitivity, nausea or vomiting, or changes of the intensity of the headache during the day.

His past medical history was significant for HIV (most recent HIV viral load 30 copies/mL, CD4 count 312 cells/μL, CD4 nadir 286 cells/μL), for which he was started on HAART with bictegravir/emtricitabine/tenofovir alafenamide 6 weeks prior to presentation. He had previously been treated for syphilis. He also had a history of tuberculosis and reported starting treatment in the Republic of the Congo that was discontinued due to medication side effects. He had recently undergone a workup for tuberculosis by an infectious disease specialist. His sputum was negative for acid-fast bacilli and *M. tuberculosis* PCR and concern for active TB was low; however, he was started on isoniazid due to the history of incomplete treatment.

His social history was notable for immigration from the Republic of the Congo to the United States 10 months ago. He also reported travel in the last month to a metropolitan area in Texas with inhalation exposure to construction dust in an old building. While he initially denied having any sexual encounters, when subsequently asked if he had touched another person recently, he reported touching a man’s penis which had active lesions on it but did not proceed to have intercourse with him.

On admission, the patient was afebrile and hemodynamically stable. He was fully alert and oriented and endorsed mild pain on palpation of his posterior neck; however, no nuchal rigidity was noted. The rest of his neurological exam, including evaluation for Kernig and Brudzinski signs, was unremarkable. Breath sounds were diminished throughout, with amphoric breath sounds appreciated at the left apex. An abdominal exam was notable for palpable splenomegaly 3 cm beneath the left costal margin. A dermatologic exam revealed dry skin on the bilateral lower extremities with areas of hyperpigmentation and some sensitivity to light touch, but no active skin or genital lesions.

**Figure 1:**
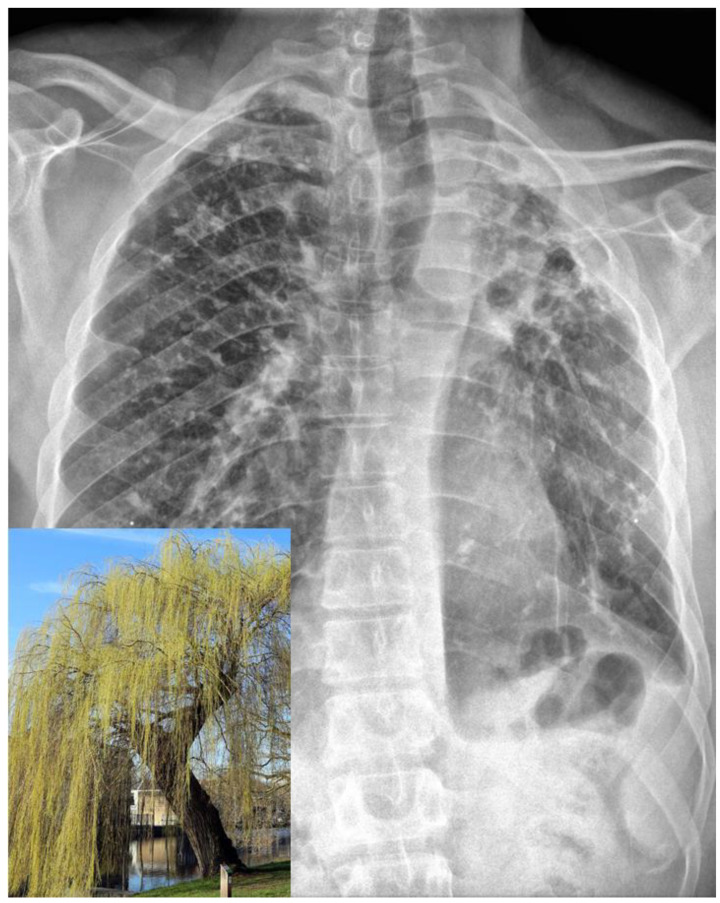
The patient’s chest X-ray exhibits the classic “weeping willow” sign within the left hemithorax, which is a distinctive feature of pulmonary scarring induced by tuberculosis. This sign results from a combination of apical contraction, bronchial drooping, and fibrotic bands that extend down to the diaphragm, creating a resemblance to a weeping willow tree. Inlet, bottom left: weeping willow tree, for comparison. Inlet image courtesy of Sailko, CC BY 3.0, via Wikimedia Commons.

An initial workup revealed a normal blood count, metabolic panel, CPK and lactate, and a mildly elevated CRP (Table 1). A chest X-ray revealed bilateral calcified granulomata, along with significant volume loss and extensive scarring in the left upper lobe with retraction of the left hilum, summarized as the classic “weeping willow” pattern, consistent with his history of extensive tuberculosis (Figure 1), unchanged from a prior X-ray.

A lumbar puncture was performed, which showed an elevated white count, normal glucose, and borderline elevated protein (Table 2). While an opening pressure was not measured, the patient reported an improvement in his headache following lumbar puncture.

Blood cultures and a multiplex CSF encephalitis/meningitis PCR panel were sent along with serologies for atypical bacterial, viral, fungal, and tick-borne illnesses.

Additional imaging studies were obtained, including a CT of his abdomen and pelvis, which was unremarkable, and a CT of his chest, which re-demonstrated tuberculosis sequela with no change from prior outpatient imaging. A brain MRI with and without contrast to assess for intracranial lesions revealed only a non-specific T2/FLAIR hyperintensity in the right cerebellar hemisphere.

Given this patient’s history of active tuberculosis and incomplete treatment, his presentation was initially concerning for tuberculosis meningitis. In addition to other common bacterial and viral causes of CNS infection, cryptococcus meningitis was also specifically of concern, as up to 10% of cryptococcal infections in patients with HIV occur with a CD4 count > 100 [4]. Other causes of HIV-related CNS disease, such as toxoplasmosis and primary CNS lymphoma, were also considered but were unlikely given that both generally appear in patients with a CD4 count < 100 [1,3]. Furthermore, brain imaging did not reveal findings concerning for either disease. Given his history of syphilis, neurosyphilis was also part of the differential. Monkeypox was considered given the patient’s history of contact with a man with active penile lesions and the rising number of cases in the community. The differential also included non-infectious etiologies of fever and headache based on the patient’s history, with isoniazid-induced aseptic meningitis and immune reconstitution inflammatory syndrome (IRIS) considered the most likely diagnoses.

Initially, IV fluids were given in the emergency department and isoniazid was held due to concern for possible drug-induced aseptic meningitis. Due to the patient’s stability, empiric antibiotics were deferred prior to lumbar puncture, and then were not started when his initial CSF studies were reassuring. He was treated supportively and monitored for the next two days. Isoniazid continued to be held for 48 h and his headaches and myalgias improved with paracetamol. He remained afebrile, did not develop any further signs/symptoms of monkeypox, and did not develop any lesions that could be unroofed for testing.

The day following admission, the patient’s encephalitis and meningitis panel, which tested for HSV 1 and 2, VZV, CMV, HHV 6, enterovirus, human parechovirus, *Cryptococcus* spp., *S. pneumoniae*, *H. influenzae*, *N, meningitides*, *L. monocytogenes*, and *E. coli*, were all negative. CSF VDRL was also negative. His preliminary blood cultures showed no growth. Serum studies revealed evidence of past infection with EBV, as the patient was positive for EBV VCA IgG and EBNA IgG, but negative for VCA IgM.

On hospital day 3, a trial of isoniazid was initiated, and the patient was monitored for 24 h with no decompensation or return symptoms. On hospital day 4, EBV PCR was positive, at 173 copies/mL. The diagnosis of aseptic meningitis in the setting of infectious mononucleosis was made, and the patient was instructed to continue to rest and take paracetamol as needed for any mild recurrent headache or myalgias.

Two weeks later, at an outpatient follow up appointment, the patient remained asymptomatic and a repeat EBV PCR was negative with 0 copies/mL detected, confirming a diagnosis of acute EBV reactivation at the time of hospitalization. He was continuing bictegravir/emtricitabine/tenofovir alafenamide, isoniazid, and pyridoxine and did not report any side effects or new symptoms from his medications.

## 3. Discussion

The present case highlights the importance of obtaining a thorough exposure history in order to guide the differential diagnosis, especially in immunocompromised hosts. The patient had multiple exposures, including recent travel, exposure to dust, and direct contact with active genital lesions, as well as prior history of HIV, tuberculosis, and syphilis. As such, the differential diagnosis was broad.

A noteworthy aspect to consider is the differential diagnosis for individuals with HIV who present with suspected CNS infection. Along with symptoms and exposures, the clinician can use the patient’s CD4 count (and nadir CD4 count) to narrow the differential. Toxoplasmosis and tuberculosis can present in patients with counts of <200 cells, while conditions such as PML or primary CNS lymphoma usually present in patients with <100 cells. Cryptococcus becomes a likely cause in patients with <50 cells. In contrast, in patients whose CD4 counts never decreased below 200, infections with any of these pathogens is unlikely [5].

CSF findings were critical in guiding the differential. Tuberculous meningitis was the primary concern but was made unlikely by the presence of normal glucose in the CSF. The initial lumbar puncture also ruled out common bacterial causes of meningitis, confirmed by the multiplex PCR. Therefore, other viral and non-infectious causes of aseptic meningitis became more likely; specifically, IRIS or isoniazid-induced aseptic meningitis.

Multiple factors led to consideration of IRIS in this patient. Latent or incompletely treated tuberculosis is among the most common opportunistic infections that increases risk for IRIS, while rapid viral suppression is a further risk factor. In this case, the patient’s viral load had decreased from 79,800 copies/mL prior to starting therapy to 30 copies/mL after one month of HAART [6]. While his presentation would have been consistent with a mild, self-limiting course of IRIS, the benefits outweighed the risks of continuing HAART.

Isoniazid-induced aseptic meningitis was considered given the patient’s recent initiation of anti-tuberculous treatment. His lymphocytic pleocytosis and mildly elevated CSF protein with negative preliminary bacterial, fungal, and mycobacterial CSF culture and PCR results were consistent with aseptic meningitis. Similar to IRIS, however, drug-induced aseptic meningitis is also a diagnosis of exclusion with no single definitive test. Isoniazid was initially held, and the patient’s symptoms resolved soon afterwards; however, when he was rechallenged with isoniazid a few days later, his symptoms did not recur, making this diagnosis unlikely.

This case is also notable for the patient’s several prominent clinical findings consistent with postprimary tuberculosis. His physical exam was notable for amphoric breath sounds, breath sounds that are produced by the flow of air through a pulmonary cavity and are reminiscent of the sound produced by blowing air in a bottle with a wide opening (such as an ancient Greek amphora). Their presence is diagnostic of an underlying cavity [7,8], while their identification at the apex, such as in this patient, is highly suggestive of a disease that causes cavitary lesions in the upper lobes, such as tuberculosis. In addition, his chest X-ray showed the presence of the classic “weeping willow” sign, characteristic of tuberculosis-induced pulmonary scarring. It is the result of a combination of apical contraction, drooping of the bronchi, and fibrotic bands reaching down to the diaphragm, which resembles the weeping willow tree [9] (Figure 1).

The growing incidence of monkeypox locally and globally and the patient’s history of contact with a partner with unknown penile lesions lead to the consideration of monkeypox as an alternative diagnosis. Myalgias, fever, and sensitivity to touch on the patient’s shins, despite a lack of dermatologic lesions on presentation, were consistent with monkeypox prodrome [10]. However, the patient’s symptoms never progressed to the development of skin lesions.

The patient’s presentation, specifically the combination of fever, myalgias, and splenomegaly, was typical of acute EBV infection. However, given his past medical history of HIV and tuberculosis, combined with his recent exposures, EBV infection was not part of the initial differential, especially given the fact that the patient had evidence of past EBV infection. However, patients with HIV are known to have episodes of EBV reactivation with active viremia [11]. In a series of 322 patients undergoing lumbar puncture for suspicion of CNS infection, EBV was detected in the CSF of 32 (9.94%) patients through PCR; an alternative diagnosis was strongly suspected in 15 (47%) of cases prior to the identification of EBV in the CSF. Among patients with positive EBV PCR, 81% were immunocompromised, 25% had a second concomitant pathogen identified in the CSF, 40.6% had encephalitis or meningoencephalitis, while 18.8% had meningitis without encephalitis [2]. Therefore, although the patient’s past medical history and recent exposures initially made acute EBV infection an unlikely diagnosis, his immunocompromised status and the potential for EBV reactivation highlight the importance of considering this pathogen in the differential diagnosis of CNS infections in patients with HIV.

## 4. Conclusions

In summary, obtaining a thorough exposure history and considering the patient’s CD4 count, CSF findings, and clinical presentation can guide the differential diagnosis in immunocompromised hosts. EBV reactivation in patients with HIV can present with atypical symptoms and should be on the differential of causes of aseptic meningitis in this population.

## Figures and Tables

**Table 1:** Laboratory parameters on admission.

Parameter	Value	Reference Value
WBC	10,400 cells/ml	4–11 × 10^3^ cells/ml
PMN	8710 cells/ml	1.2–9.3 × 10^3^ cells/ml
Hb	14.6 g/dl	13.5–17.5 g/dL
PLT	286,000 cells/ml	150–350 × 10^3^ cells/ml
Na	139 mmol/L	137–145 mmol/L
K	4.3 mmol/L	3.5–5.1 mmol/L
HCO3	23 mmol/L	22–30 mmol/L
BUN	14 mg/dL	9–20 mg/dL
Cr	1.2 mg/dL	0.7–1.3 mg/dL
CPK	144 U/L	55–170 U/L
CRP	30.6 mg/L	<10.0 mg/L
Lactate	1 mmol/L	0.7–2.1 mmol/L

**Table 2:** Lumbar puncture results.

CSF	Tube 1	Tube 4	Reference Range
Color	Pink	Colorless	Colorless
Character	Clear	Slightly Cloudy	Clear
Xanthochromia	Absent	Absent	Absent
WBC	27	37	0–5 10^3^/uL
Differential	PMN 52%, LY 48%	PMN 73%, LY 22%, MONO 5%	PMN 0–5%, LY 0–65%, MONO 0–30%
RBC	4000	7	0–5 cells/CCM
Protein	-	64	12–60 mg/dl
Glucose	-	54	40–70 mg/dl

## Data Availability

All data pertaining to the current work are included within this manuscript.
